# Epidemiology of more than 50,000 ankle fractures in the Swedish Fracture Register during a period of 10 years

**DOI:** 10.1186/s13018-023-03558-2

**Published:** 2023-01-31

**Authors:** Emilia Möller Rydberg, David Wennergren, Caroline Stigevall, Jan Ekelund, Michael Möller

**Affiliations:** 1 Department of Orthopedics, Institute of Clinical Sciences, Sahlgrenska Academy, University of Gothenburg, Gothenburg, Sweden; 2 Department of Orthopedics, Sahlgrenska University Hospital, Göteborgsvägen 31, 431 80 Gothenburg/Mölndal, Sweden; 3 Centre of Registers Västra Götaland, Gothenburg, Sweden

**Keywords:** Epidemiology, Ankle fracture, Orthopaedic surgery, The Swedish Fracture Register

## Abstract

**Background:**

Despite being one of the most common types of fracture, there is a lack of epidemiological studies involving ankle fractures of all kinds. Since 2012, the Swedish Fracture Register (SFR) has prospectively collected data on surgically and non-surgically treated ankle fractures. The aim of this study is to describe the epidemiology of ankle fractures between 2012 and 2022.

**Methods:**

All ankle fractures registered in the SFR between 1 April 2012 and 31 March 2022 in patients aged 16 years or older were included. Information on age, sex, mechanism of injury, fracture classification according to AO/OTA and high- or low-energy trauma was retrieved from the SFR.

**Results:**

During the study period, 56,439 patients sustained 57,443 ankle fractures. Women (61%) were more commonly affected than men (39%). The mean age at the time of injury was 55 years. Men were found to be younger at the time of injury compared with women. Women were found to sustain open fractures more frequently, whereas the men more frequently sustained high-energy trauma. The most common mechanism of injury for all ankle fractures and for each AO/OTA44 fracture group separately was a simple fall. A seasonal variation in ankle fractures was found, where the number of ankle fractures peaked during the between November and March.

**Conclusions:**

This study presents the epidemiology of all AO/OTA types of ankle fractures. We have demonstrated that most ankle fractures are caused by a simple fall and occur during wintertime. Women are more commonly affected than men and are older at the time of injury. These findings indicate that age-related skeletal fragility, as well as an increasing risk of simple falls in the elderly, may be risk factors for ankle fractures. This study will contribute to the planning of primary prevention for ankle fractures.

## Background

Ankle fractures are the third most common type of fracture, affecting both sexes and all age groups [1–3]. The spectrum of ankle fractures ranges from simple avulsions that can be safely treated non-surgically without follow-up to complex, open injuries that require multiple surgeries and long-term rehabilitation [4]. An increasing incidence of ankle fractures, especially in the elderly, has been reported in several studies over the past few years [5–8]. Despite this, there is a lack of up-to-date, comprehensive epidemiological studies including all kinds of ankle fracture.

The Swedish Fracture Register (SFR) is a national quality register that prospectively collects data on all fractures treated by orthopaedic surgeons since 2011 [9, 10]. Ankle fractures have been registered in the SFR since April 2012. A recent validity study by Bergdahl et al*.* deemed the SFR to be a “complete, accurate and efficient source of information” for epidemiological studies [11]. Fractures are registered in the SFR by residents or orthopaedic surgeons and classified according to the AO/OTA 2007 (Arbeitsgemeinschaft für Osteosynthesefragen/Orthopaedic Trauma Association) classification system [12–14]. Several studies have been conducted showing substantial accuracy and high reliability of fracture classification in the SFR for a number of injury locations, including ankle fractures [15–17].

Studies have indicated findings of seasonal variations in ankle fractures, as well as sex and age, influencing the type of fracture sustained [18, 19]. However, most previous studies have only reported on hospitalised or surgically treated patients [20–22]. Since a substantial proportion of ankle fractures are neither surgically treated nor hospitalised, the full picture of ankle fracture epidemiology remains unclear. Large epidemiological studies are therefore needed fully to elucidate the underlying injury mechanisms, the demographics of each fracture group and the influence of sex and age on the sustained fracture. The aim of the present study is to describe the epidemiology of AO/OTA-classified ankle fractures in a large cohort of consecutive fractures collected in the SFR over a ten-year period.

## Materials and methods

### Study design and setting

The current study is an observational register study based on data from the SFR. The SFR prospectively collects data on fractures of all types (non-surgically and surgically treated), including data on injury mechanism, type of fracture and subsequent treatment method [9]. Since the inception of the SFR, the number of participating orthopaedic departments has gradually increased, with full coverage, i.e. all 54 orthopaedic departments in Sweden are enrolled, since 2020 [10].

### Patient selection and study variables

**Fig. 1:**
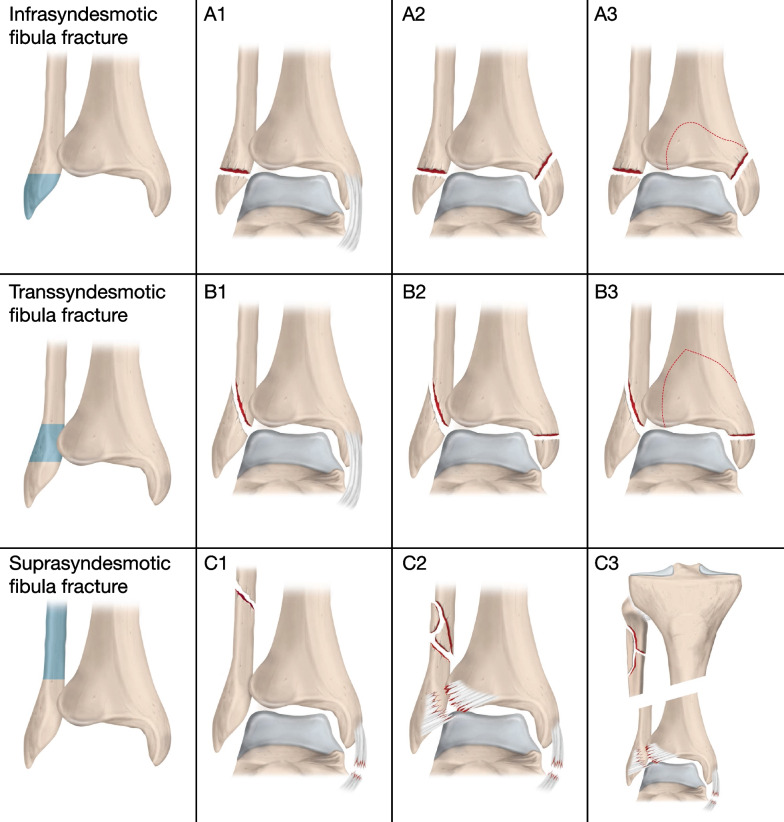
The AO/OTA classification of ankle fractures. Illustration by Pontus Andersson/Pontus Art Production

This study comprised all ankle fractures (AO/OTA44; Fig. 1) in adults (16 years and older) registered in the SFR at all participating orthopaedic departments in Sweden during a ten-year period between 1 April 2012 and 31 March 2022. Only patients with a Swedish personal identification number and fractures occurring in Sweden are registered in the SFR and are therefore included in the study.

Epidemiological data on age, sex, injury date, injury mechanism, injury type (high- or low-energy trauma), fracture classification and chosen type of treatment (surgical or non-surgical) were retrieved from the SFR. Information regarding fracture classification included the fracture type and group according to the AO/OTA classification, as well as side and open or closed fracture. For injury mechanisms, the same six categories (simple fall, fall from a height, unspecified fall, traffic, miscellaneous and non-traumatic) defined by Bergdahl et al*.* and Wennergren et al*.* were used [23, 24].

### Statistics

Descriptive statistics for categorical data were presented as the count and proportion (%) and for continuous data as the mean (SD) and median (range). Statistical analyses were performed using IBM SPSS Statistics v27.

## Results

During the ten-year study period, 56,439 patients sustained a total of 57,443 ankle fractures. Bilateral fractures on the same injury occasion were seen in 156 (0.3%) of the patients and 845 (1.5%) patients sustained more than one ankle fracture (on different injury occasions) during the study period. Of the patients who had more than one fracture during the study period (bilateral fractures not included), 364 patients sustained another fracture to the same ankle and 481 patients fractured the contralateral ankle.

During the last full year of the study, 2021, when the SFR had 100% coverage, 8762 ankle fractures occurred in individuals ≥ 18 years. The same year, the SFR had a 70% completeness in ankle fracture registrations and Sweden had 8 254 086 inhabitants ≥ 18 years of age [25, 26]. This represents an estimated incidence of ankle fractures in Sweden in 2021 of approximately 152/100,000 persons per year.

### Fracture classification

Of the 56,414 fractures in the present study, 13,685 (24.3%) were classified as AO/OTA type A, 35,892 (63.6%) as AO/OTA type B and 6837 (12.1%) as AO/OTA type C (Table 1). Within the three AO/OTA types (A, B and C), fractures in group 1 (A1, B1, C1) dominated in all three types, respectively. In the cohort of A-type fractures, the A1 group accounted for 69%, whereas only 6% A3 fractures were seen. Within the B-type cohort, 52% were B1 fractures (25% B2 and 23% B3). For C-type fractures, 45% were classified as C1, 27% as C2 and 29% as C3 (Table 1). Another 953 fractures were classified as “not able to classify”, and 76 fractures were classified as paediatric fractures.

**Table 1:** Demographics of patients with ankle fractures, stratified by AO/OTA fracture types and groups

		Type A *n* = 13,685 (24.3%)			Type B *n* = 35,892 (63.6%)			Type C *n* = 6837 (12.1%)		
	All fractures^a^ *n* = 57,443	A1 (*n* = 9,428; 68.9%)	A2 (*n* = 3,389; 24.8%)	A3 (*n* = 868; 6.3%)	B1 (*n* = 18594; 51.8%)	B2 (*n* = 9069; 25.3%)	B3 (*n* = 8229; 22.9%)	C1 (*n* = 3056; 44.7%)	C2 (*n* = 1818; 26.6%)	C3 (*n* = 1963; 28.7%)
% of all ankle fractures		16.4%	5.9%	1.5%	32.4%	15.8%	14.3%	5.3%	3.2%	3.4%
Sex, *n* (%)										
Male Female	22,653 (39) 34,790 (61)	2989 (32) 6439 (68)	1629 (48) 1760 (52)	350 (40) 518 (60)	7921 (43) 10,673 (57)	3365 (37) 5704 (63)	2274 (28) 5955 (72)	1500 (49) 1556 (51)	875 (48) 943 (52)	1209 (62) 754 (38)
Age,										
mean (range)										
Total Male Female	55 (16 to 107) 50 (16 to 101) 58 (16 to 107)	54 (16 to 104) 48 (16 to 94) 57 (16 to 104)	52 (16 to 106) 45 (16 to 96) 58 (16 to 106)	51 (16 to 98) 46 (16 to 94) 54 (16 to 98)	55 (16 to 104) 52 (16 to 101) 58 (16 to 104)	58 (16 to 104) 52 (16 to 99) 61 (16 to 104)	59 (16 to 107) 53 (16 to 98) 61 (16 to 107)	52 (16 to 99) 47 (16 to 94) 57 (16 to 99)	49 (16 to 101) 46 (16 to 96) 52 (16 to 101)	53 (16 to 98) 50 (16 to 98) 57 (16 to 98)
Fracture^b^, *n* (%)										
Open High energy Low energy	1023 (1.8) 2674 (4.7) 45,755 (79.7)	29 (0.3) 287 (3.0) 7545 (80.0)	60 (1.8) 453 (13.4) 2372 (70.0)	13 (1.5) 61 (7.0) 657 (75.7)	73 (0.4) 460 (2.5) 15,452 (83.1)	305 (3.4) 454 (5.0) 7144 (78.8)	324 (3.9) 347 (4.2) 6696 (81.4)	78 (2.6) 195 (6.4) 2285 (74.8)	98 (5.4) 209 (11.5) 1328 (73.0)	16 (0.8) 109 (5.6) 1596 (81.3)

Fractures that were classified as “not able to classify” and paediatric fractures are included in the total numbers in the “All fractures” column

Row number two displays the number and percentage of cases for each AO/OTA group within that AO/OTA type

^a^953 fractures were classified as “not able to classify” and 76 fractures were classified as paediatric fractures and they are included here in the total number

^b^For high- and low-energy injuries, data are not shown for cases where this variable was registered as “unknown” or “not applicable”

### Demographics

For all ankle fractures, women (61%) were more commonly affected than men (39%; Table 1). For all ankle fractures, the mean age at the time of sustaining the fracture was 55 years and ranged from 16 to 107 years. For the different AO/OTA groups, the lowest mean age was found in the C2 group with 49 years at the time of injury, while the highest age was found in the B3 group with a mean age of 59 years. Men were found to be younger at the time of injury (mean age, 50 years; range 16–101) compared with the women who had a higher mean age of 58 years (range 16–107). The patients with fractures that were classified as “not able to classify” had a more even distribution between the sexes, a slightly lower mean age and a slightly higher proportion of high-energy injuries and open fractures than the group comprising all ankle fractures, comparable to the fractures classified as A2, C1 or C2.

**Fig. 2:**
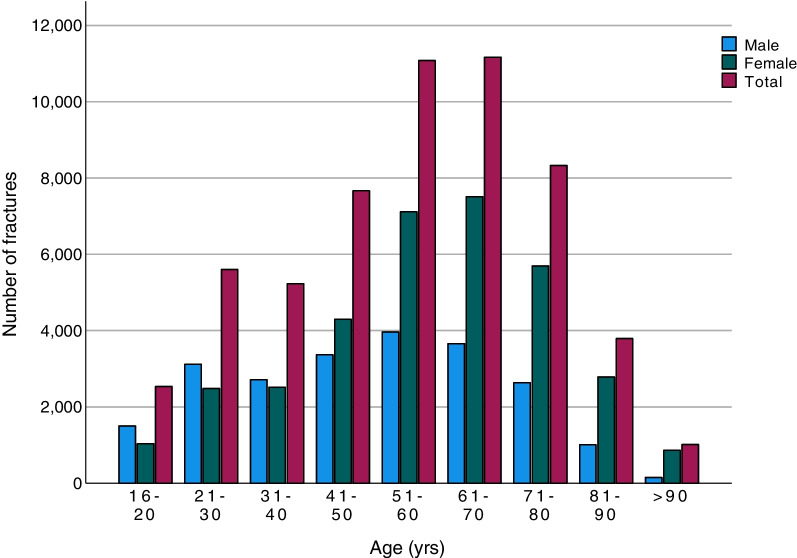
Distribution between the sexes for ankle fractures by age group. Paediatric fractures and fractures that were registered as “not able to classify” are not included. Data shown for 1 April 2012 to 31 March 2022

The age and sex distribution for all ankle fractures demonstrated that the frequency of ankle fractures peaks in total numbers between the ages of 50 and 70 years (Fig. 2). Only in the age groups below 40 do men have a higher frequency of ankle fractures than women (Fig. 2).

### Fracture characteristics

**Fig. 3:**
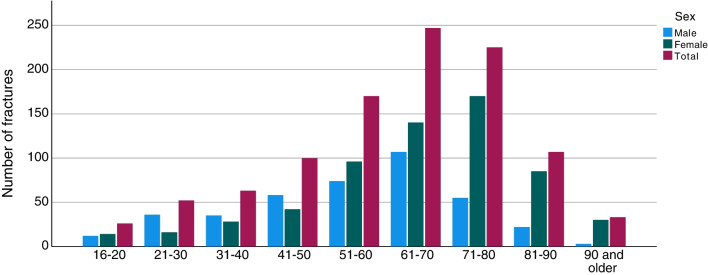
Age and sex distribution of open ankle fractures. Data shown for 1 April 2012 to 31 March 2022

**Fig. 4:**
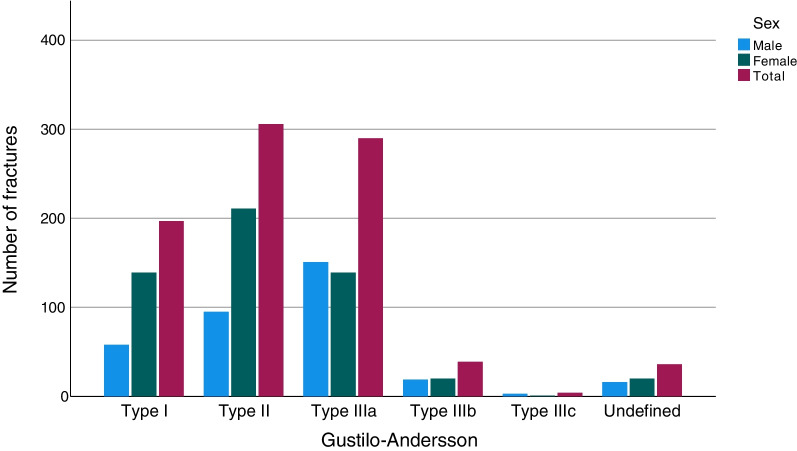
Gustilo–Andersson grade of open ankle fractures. Data shown for 1 April 2012 to 31 March 2022

**Fig. 5:**
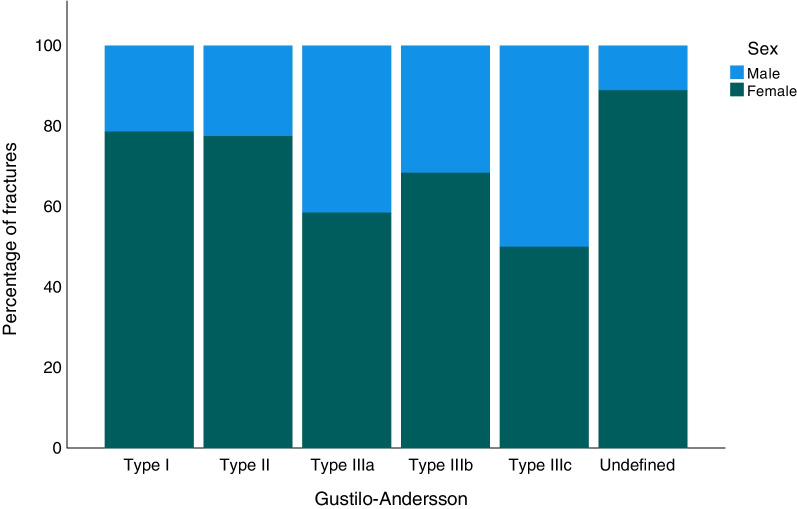
Gustilo–Andersson grade of open ankle fractures in patients over the age of 65 years. Data shown for 1 April 2012 to 31 March 2022

**Fig. 6:**
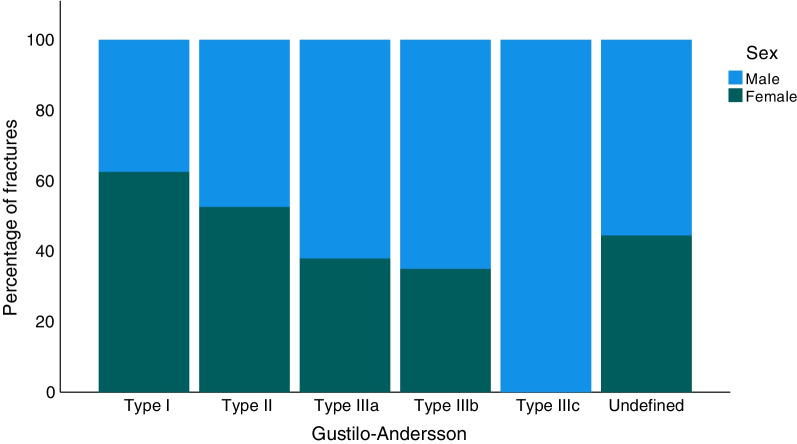
Gustilo–Andersson grade of open ankle fractures in patients under the age of 65 years. Data shown for 1 April 2012 to 31 March 2022

Of all the ankle fractures in the study, 1.8% were open (*n* = 1023; Table 1). When analysed for AO/OTA fracture group, open fractures were most frequent in the C2 group with 5.4% (*n* = 9), while the A1 group had the fewest open fractures, only 0.3% (*n* = 29; Table 1). Open fractures were most common between the ages of 50 and 80 years (Fig. 3). In all the age groups over 60 years, open fractures were more common in women than men, whereas, in the age groups of 21–50, the men were more commonly affected. Open fractures of Gustilo–Andersson type II (wound > 1 cm) were most common and 2/3 of this group consisted of women (Fig. 4). For patients over the age of 65 years women dominated in all Gustilo–Andersson injury types (Fig. 5). For patients under the age of 65 years, Gustilo–Andersson injuries types I and II were dominated by women, whereas men dominated the Type III injuries (Fig. 6).

**Fig. 7:**
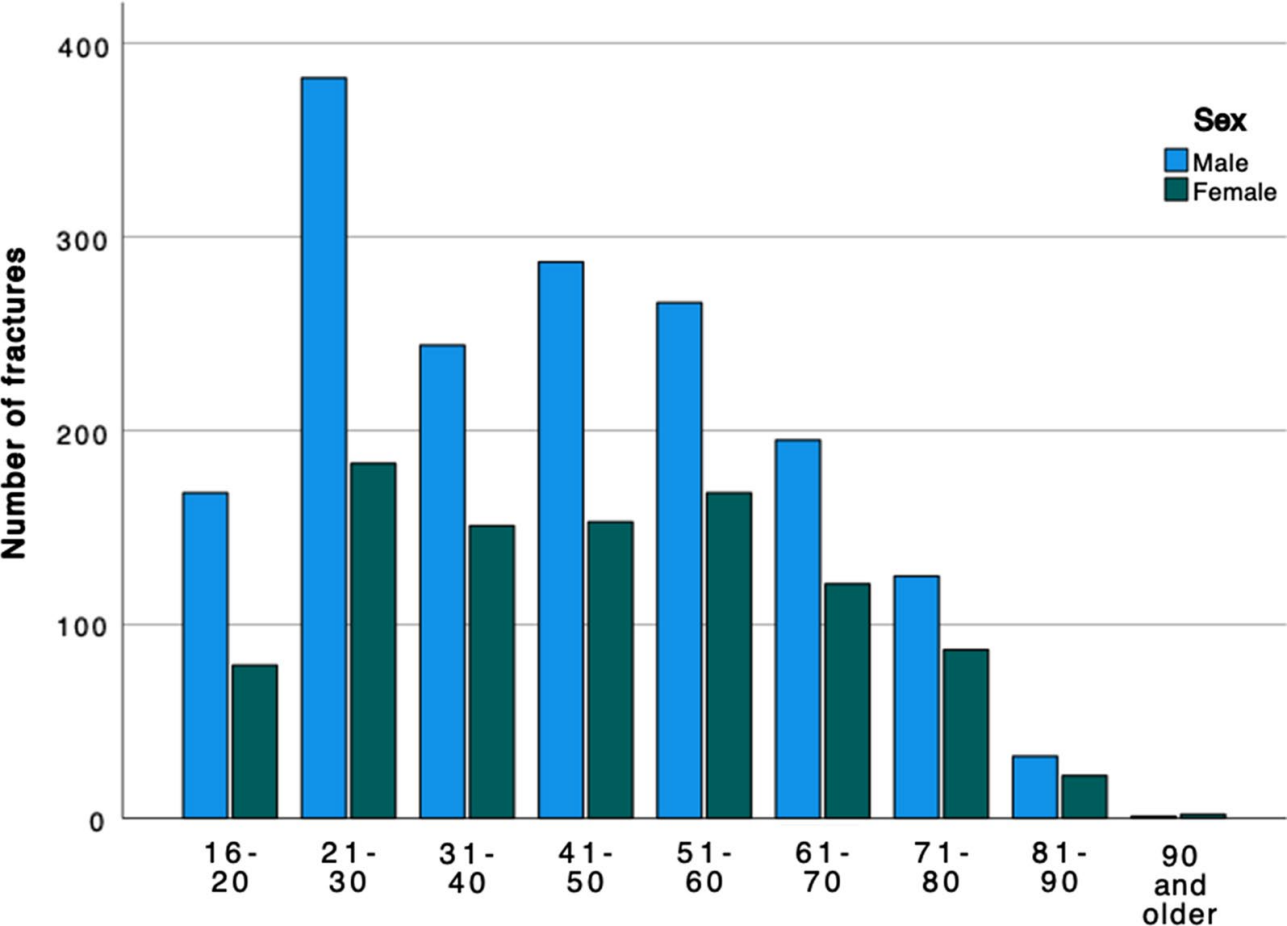
Age and sex distribution of ankle fractures sustained in high-energy trauma. Data shown for 1 April 2012 to 31 March 2022

High-energy trauma was the underlying cause of 4.7% (*n* = 2674) of all the ankle fractures in the study. The AO/OTA-A2 group had the highest proportion of high-energy trauma cases, whereas the AO/OTA-B1 group had the fewest high-energy trauma cases (2.5%, *n* = 460). High-energy trauma was most common in the age group of 21–30 years. In all age groups, men were more frequently injured by high-energy trauma than women (Fig. 7).

### Mechanism of injury

The most common mechanism of injury for all ankle fractures and for each AO/OTA44 fracture group separately was a simple fall (Tables 2, 3). Traffic injuries comprised a substantially higher proportion of high-energy trauma (29.2%) and resulted more frequently in open fractures (4.7%) than other injury mechanisms. Miscellaneous injuries (fractures with a mechanism of injury that did not fit the other categories, such as sports injuries and fractures sustained in fights) and traffic injuries were associated with male sex and a lower mean age than the other injury mechanisms.

**Table 2:** Frequency of open fractures and high-energy injuries for all ankle fractures^a^, in correlation to mechanism of injury

	Mechanism of injury					
	Simple fall	Fall from a height	Unspecified fall	Traffic	Miscellaneous^b^	Non-traumatic^c^
Number of fractures	37,674	6058	3788	4419	3115	289
%	65.7	10.6	6.6	7.7	5.4	0.5
Open						
*n*	503	165	53	206	77	0
%	1.3	2.7	1.4	4.7	2.5	0
High energy						
*n*	339	562	53	1,289	397	0
%	0.9	9.3	1.4	29.2	12.7	0
Mean age						
Years	57	54	57	47	44	57
Range	16–106	16–99	16–107	16–97	16–98	16–92
Female sex						
%	63.9	59.9	60.8	49.1	36.2	72.7

Number of fractures where data on mechanism of injury were missing: 2024 (3.5%)

^a^76 fractures were classified as paediatric fractures, and they are not included in this analysis

^b^Miscellaneous injuries include fractures with a mechanism of injury that did not fit the other categories, such as sports injuries and fractures sustained in fights

^c^Non-traumatic fractures include pathological fractures, spontaneous fractures and stress fractures

**Table 3:** Mechanism of injury for each AO/OTA44 ankle fracture group^a^

Mechanism of injury, *n* (%)	A1	A2	A3	B1	B2	B3	C1	C2	C3
Simple fall	6267 (66.5)	1540 (45.4)	495 (57.0)	12,877 (69.3)	6190 (68.3)	5677 (69.0)	1852 (60.6)	1049 (57.7)	1299 (66.2)
Fall from a height	932 (9.9)	492 (14.5)	118 (13.6)	1728 (9.3)	868 (9.6)	995 (12.1)	354 (11.6)	235 (12.9)	208 (10.6)
Unspecified fall	701 (7.4)	206 (6.1)	68 (7.8)	1,255 (6.7)	614 (6.8)	481 (5.8)	186 (6.1)	86 (4.7)	120 (6.1)
Traffic	553 (5.9)	599 (17.7)	89 (10.3)	1,159 (6.2)	675 (7.4)	585 (7.1)	251 (8.2)	236 (13.0)	161 (8.2)
Miscellaneous^b^	529 (5.6)	403 (11.9)	57 (6.6)	851 (4.6)	379 (4.2)	245 (3.0)	244 (8.0)	157 (8.6)	118 (6.0)
Non-traumatic^c^	36 (0.4)	16 (0.5)	6 (0.7)	99 (0.5)	9 (0.1)	5 (0.1)	71 (2.3)	1 (0.1)	0 (0)
Data missing	410 (4.3)	133 (3.9)	35 (4.0)	625 (3.4)	334 (3.7)	241 (2.9)	98 (3.2)	54 (3.0)	57 (2.9)

^a^953 fractures were classified as “not able to classify” and 76 fractures were classified as paediatric fractures and they are not included

^b^Miscellaneous injuries include fractures with a mechanism of injury that did not fit the other categories, such as sports injuries and fractures sustained in fights

^c^Non-traumatic fractures include pathological fractures, spontaneous fractures and stress fractures

### Seasonal variation

**Fig. 8:**
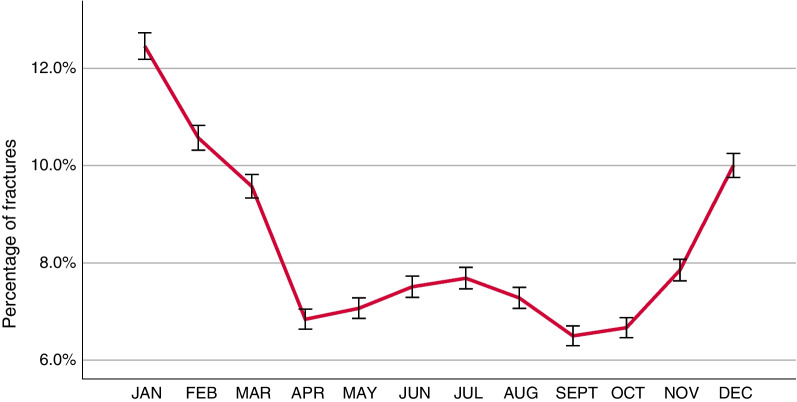
Seasonal variation in ankle fractures. Error bars: 95% CI

**Fig. 9:**
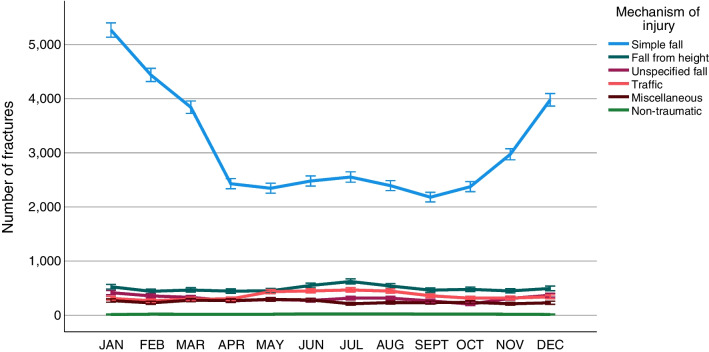
Seasonal variation in the number of ankle fractures according to mechanism of injury. Error bars: 95% CI

**Fig. 10:**
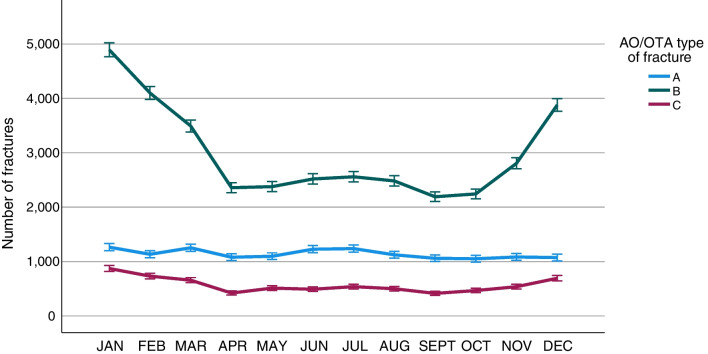
Seasonal variation in the number of ankle fractures for each fracture type, respectively. Error bars: 95% CI

A notable seasonal variation in ankle fractures was found. The number of ankle fractures was found to peak during the Swedish winter months (November to March) and to decline in spring and summer (Fig. 8). The seasonal variation in fractures was found to be driven by fractures sustained by simple falls (Fig. 9). When analysed by fracture type, the same seasonal variation was seen in B-type fractures but not for the other fracture types (Fig. 10).

## Discussion

In this ten-year epidemiological study of more than 50,000 ankle fractures, we have shown that the majority of ankle fractures affect women and are caused by a simple fall. Affected men are younger at the time of the injury and are more often injured by a high-energy trauma. Open ankle fractures, on the other hand, are most common in women over the age of 60.

Ankle fractures are the third most common fracture type, and numerous studies have shown that the incidence of ankle fractures is increasing [7, 8, 19, 27]. In spite of this, there are few exhaustive epidemiological studies. Recent studies from the Nordic countries have indicated seasonal variations in fractures of the humerus, proximal femur and ankle [21, 24, 27, 28]. The findings in the present study further confirm these findings, as a distinct seasonal variation in ankle fractures is demonstrated, with a peak in fractures between November and March. In the present study, we have further demonstrated that the seasonal variation is the result of simple falls causing B-type fractures. As stipulated in the above-mentioned studies, this is probably due to a large extent to snow and ice causing slippery conditions and a high risk of simple falls [21, 24, 27, 28]. Simple falls are the most common injury mechanism behind ankle fractures in general and are also pronounced in B-type fractures. Elsoe et al*.* attributed their findings of seasonal variations in ankle fractures to exceptionally snowy and icy winters during the study period, but, in the present study, we have presented a similar seasonal variation during a study period of ten years. As the present study is both multicentre and spans a ten-year period, variations in single years or single locations within Sweden will most probably not have had an impact on the results. So, the demonstrated seasonal variation in ankle fractures appears to be consistent, at least in Nordic conditions. It would have been interesting to compare these findings with those in similar studies from countries with a different climate, without snowy and icy winters, but to our knowledge no studies of seasonal variations in ankle fractures from outside Europe can be found.

B-type fractures constitute almost two-thirds of all ankle fractures, are the fracture type with the highest mean age at the time of injury and are also a fracture type that predominantly affects women. A recent systematic review of the association between low bone mineral density (BMD) and ankle fractures in elderly patients has identified a significant association between ankle fractures and low BMD in the femoral neck [29]. Despite being outside the scope of the current study, our findings of a high mean age at the time of injury for simple falls, together with a predominance of women, support the hypothesis that ankle fractures in the elderly could be regarded as an osteoporotic fracture type. Together with the findings of seasonal variations in simple falls and B-fractures, this suggests that measures should be taken to limit the risk of falls in the elderly during wintertime to reduce the incidence of ankle fractures.

In the current study, 1.8% of all ankle fractures were found to be open. This correlates well to the findings in both other recent studies and older studies like Court-Brown et al*.* from 1998 [2, 8, 19, 30]. The present study demonstrates that women sustain an open ankle fracture more frequently than men, thereby confirming the findings of Bugler et al*.* [30]. Open ankle fractures appear to affect young men and older women. The extent of open injuries, graded by Gustilo–Andersson, demonstrates that grade II injuries were most common. Gustilo–Andersson injuries of grades I-II were shown predominantly to affect women, whereas injuries of Gustilo–Andersson type IIIa were more often seen in men. Group IIIa comprises open fractures with adequate soft tissue coverage despite extensive soft tissue damage, but this group also comprises all high-energy cases, regardless of the size of wound. As discussed above, men are more frequently injured in high-energy injuries, which explains this finding. To conclude, open fractures of grades I and II are seen in women above the age of 60 and more severe open fractures (grade III) are more common in younger men.

High-energy trauma was the underlying cause of 4.7% of all the ankle fractures in this study, in line with the findings of another recent study by Bergh et al*.* [2]. In the current study, traffic injuries were shown to comprise the highest proportion of open fractures (4.7%) and high-energy trauma is the underlying cause in almost 30% of cases. Predominantly men were involved in traffic injuries and miscellaneous injuries, including sports injuries, injuries by tools and machines, as well as assaults. These findings correlate well with the findings of Wennergren et al*.* relating to tibial fractures [23]. The findings relating to the underlying injury mechanisms for ankle fractures presented in this study correlate well with the findings of Thur et al*.* and Vieira Cardoso et al*.* [20, 22]. In these studies, a slightly higher proportion of open fractures (3% and 8%, respectively) were found, which is understandable given that only hospitalised and surgically treated patients, respectively, were studied.

The SFR offers a unique opportunity to conduct large epidemiological studies. The classification of ankle fractures, as well as other types of fracture, in the SFR has been validated and shown to have substantial accuracy [15, 17, 31]. Compared with data from the Swedish National Patient Register (NPR), the SFR has been shown to constitute a complete and accurate source of information for epidemiological studies [11]. To our knowledge, this is the largest, most comprehensive epidemiological study of ankle fractures ever conducted. One of the strengths of the current study is that it is a multicentre study that includes ankle fractures of all kinds, treated both surgically and non-surgically and hospitalised and non-hospitalised. Another strength of this study is the length of the time period studied, i.e. ten years, which reduces the risk of variations in single years affecting the results.

One limitation of this study is the continuous expansion of the SFR during the study period. As the number of participating departments has increased over the years, more fractures are being registered in the SFR and more substantiated conclusions can be drawn from the data. However, the increasing number of participating departments during the study period made calculations of incidence in this study difficult to perform. Nevertheless, by the end of the study period, the SFR comprised all the orthopaedic departments in Sweden and a calculation of the estimated incidence of ankle fractures in Sweden in 2021 was deemed possible to conduct and revealed an incidence comparable to other recent studies.

## Conclusions

This study presents the epidemiology of all AO/OTA types of ankle fractures in Sweden over a ten-year period. We have shown that most ankle fractures are caused by a simple fall, affect women and occur during wintertime. These findings indicate that age-related skeletal fragility, as well as an increasing risk of simple falls in the elderly, may be risk factors for ankle fractures. The current study demonstrates that men sustain their ankle fractures at a younger age and are more frequently injured by a high-energy trauma. As further shown, open ankle fractures are most common in women over the age of 60, but the severe open injuries more frequently affect men. This study will contribute to the planning of primary prevention for ankle fractures and will be of value for the distribution of healthcare resources.

## Data Availability

The datasets used and/or analysed during the current study are available from the corresponding author on reasonable request.

## Abbreviations

SFR: The Swedish Fracture Register
AO/OTA: Arbeitsgemeinschaft für Osteosynthesefragen/Orthopaedic Trauma Association
SD: Standard deviation
BMD: Bone mineral density
NPR: The Swedish National Patient Register
